# Dynamic Interactions Between the Genome and an Endogenous Retrovirus: *Tirant* in *Drosophila simulans* Wild-Type Strains

**DOI:** 10.1534/g3.118.200789

**Published:** 2019-01-18

**Authors:** Marie Fablet, Angelo Jacquet, Rita Rebollo, Annabelle Haudry, Carine Rey, Judit Salces-Ortiz, Prajakta Bajad, Nelly Burlet, Michael F. Jantsch, Maria Pilar García Guerreiro, Cristina Vieira

**Affiliations:** * Laboratoire de Biométrie et Biologie Evolutive, Université de Lyon; Université Lyon 1; CNRS; UMR 5558, Villeurbanne 69622, France; † BF2i, Univ Lyon, INSA-Lyon, INRA, UMR 0203, Villeurbanne 69621, France; ‡ Laboratoire de Biologie et Modélisation de la Cellule, UnivLyon, ENS de Lyon, Univ Claude Bernard, CNRS UMR 5239, INSERM U1210, Lyon 69007, France; § Center for Anatomy and Cell Biology, Division of Cell- and Developmental Biology, Medical University of Vienna, Vienna 1090, Austria; ** Grup de Genòmica, Bioinformàtica i Biologia Evolutiva, Departament de Genètica i Microbiologia, Universitat Autònoma de Barcelona, Bellaterra-Barcelona 08193, Spain

**Keywords:** Transposable element, Retrotransposon, *Drosophila*, epigenetic control, piRNA, chromatin

## Abstract

All genomes contain repeated sequences that are known as transposable elements (TEs). Among these are endogenous retroviruses (ERVs), which are sequences similar to retroviruses and are transmitted across generations from parent to progeny. These sequences are controlled in genomes through epigenetic mechanisms. At the center of the epigenetic control of TEs are small interfering RNAs of the piRNA class, which trigger heterochromatinization of TE sequences. The *tirant* ERV of *Drosophila simulans* displays intra-specific variability in copy numbers, insertion sites, and transcription levels, providing us with a well-suited model to study the dynamic relationship between a TE family and the host genome through epigenetic mechanisms. We show that *tirant* transcript amounts and piRNA amounts are positively correlated in ovaries in normal conditions, unlike what was previously described following divergent crosses. In addition, we describe *tirant* insertion polymorphism in the genomes of three *D. simulans* wild-type strains, which reveals a limited number of insertions that may be associated with gene transcript level changes through heterochromatin spreading and have phenotypic impacts. Taken together, our results participate in the understanding of the equilibrium between the host genome and its TEs.

All genomes contain repeated sequences that are known as transposable elements (TEs). These are sequences that can move and multiply along the chromosome arms, generating most of the time deleterious mutations. A few decades ago, TEs were only acknowledged as parasitic “junk DNA”; however, we now have evidence that host-TE relationships may range from parasitism to mutualism, as do all symbiotic interactions (Biémont and Vieira 2006; Jangam *et al.* 2017). TEs may be of various structures (Wicker *et al.* 2007), among which are endogenous retroviruses (ERVs). ERVs are sequences similar to retroviruses and are transmitted across generations from parent to progeny. Due to the potential harmful activity of TEs, mechanisms that allow to control them have been favored by natural selection, and consist mainly of epigenetic processes (Slotkin and Martienssen 2007; Siomi *et al.* 2011). In *Drosophila*, knowledge of such processes is increasingly accumulating. At the center of the epigenetic control of TEs are small interfering RNAs of the piRNA class, which trigger heterochromatinization of TE sequences through trimethylation of histone 3 lysine 9 (H3K9me3) (Sienski *et al.* 2012; Le Thomas *et al.* 2013).

TE impacts on genomes have varied nature and extents, from deleterious to adaptive (reviewed in Cordaux and Batzer 2009; Casacuberta and González 2013; Fablet *et al.* 2017). For instance, TE insertions may cause diseases (Hancks and Kazazian 2016), whereas in some cases, TEs may be recruited by the host genome and perform endogenous functions. The most famous example is the *syncytin* gene in mammals, which is an ancient TE gene and is now essential for placenta formation (Mi *et al.* 2000). TEs may also be involved in adaptation. A*s* an example, the *Accord* TE inserted into the 5′ end of the *Cyp6g1* gene of *Drosophila melanogaster* is found in some strains and provides them with increased resistance to insecticides (Daborn *et al.* 2002).

Despite their potential harmful impacts, TEs may reach high proportions of genome sequences (Biémont and Vieira 2006; Tenaillon *et al.* 2010; Elliott and Gregory 2015). Therefore, it is of fundamental interest to precisely understand TE dynamics within genomes, especially at the transcriptional level, which is the preliminary stage before mobilization. In this study, we propose to use the *tirant* element of *Drosophila simulans* to tackle this important issue.

*Tirant* belongs to the ERV class of retrotransposons (Terzian *et al.* 2001). It is made of three open reading frames (ORFs), namely *gag*, *pol*, and *env*, which provide all proteins necessary to fulfill the retroviral cycle, and is bordered by two long terminal repeats (LTRs), which include regulatory signals. Two subfamilies can be distinguished based on their sequence similarity and the structure of a minisatellite located in the 5′ untranslated region (UTR) (Fablet *et al.* 2006). Subfamily C is responsible for *tirant* activity while subfamily S is heterochromatic, low-copy-number, and silent (Fablet *et al.* 2006, 2009). *Tirant* was first discovered in *D. melanogaster* (Garrell and Modolell 1990) and found to occur in related species (Fablet *et al.* 2007). Its number of copies and insertion sites, together with expression profiles vary across *D. simulans* wild-type strains (Vieira *et al.* 1999; Fablet *et al.* 2006; Akkouche *et al.* 2012), providing a powerful system to investigate the impacts of ERVs on the host genome, particularly regarding neighboring gene expression and epigenetic signatures.

The analyses of RNA-seq and small RNA-seq data indicate that *tirant* transcript amounts and piRNA amounts are positively correlated in ovaries in normal conditions, while they were previously found to be negatively correlated in some divergent crosses leading to *tirant* deregulation. These results allow us to propose that the shape of the correlation between the amounts of TE transcripts and the corresponding piRNAs are indicative of genome stability. In addition, we describe *tirant* insertion polymorphism in the genomes of three *D. simulans* wild-type strains, which reveals a limited number of insertions that may be associated with gene transcript level changes through heterochromatin spreading and have phenotypic impacts. Taken together, our results participate in the understanding of the equilibrium between the host genome and its TEs.

## Material and Methods

### Drosophila stocks

*Drosophila* strains were maintained in the laboratory at 24° as small-mass cultures. Chicharo, Mayotte, Makindu and Zimbabwe are wild-type strains, anciently sampled from the field; w501 is the major strain of the *D. simulans* sequenced genome.

### Small RNA analysis

We used small RNA-seq data obtained from ovaries from Akkouche *et al.* (2013) and Lerat *et al.* (2017), corresponding to the Chicharo, Mayotte and Makindu strains. We first removed adapter sequences using cutadatp (Martin 2011) -a CTGTAGGCACCATCAA. Using PRINSEQ lite version 0.20.4 (Schmieder and Edwards 2011), we filtered reads of size 23 to 30 nt and considered these as piRNAs. Using a modified version of the TEcount module of TEtools (Lerat *et al.* 2017), we mapped the reads against a list of TE sequences either in the sense or antisense directions. The list of TE sequences is made of the TE insertions retrieved from *D. simulans* sequenced genome (Lerat *et al.* 2017). Read count numbers were normalized to miRNA read count numbers (miRNA sequences retrieved from FlyBase: dsim-all-miRNA-r2.02.fasta.gz).

We looked for ping-pong signatures using signature.py with the options min_size = 23 and max_size = 30 (Antoniewski 2014). We used as input files SAM alignments obtained from cleaned small RNA reads against our C-subfamily complete (8.5 kb) reference *tirant* sequence: accession number AC0054444, positions 50,203 to 58,729, extracted from the *D. melanogaster* genome, as already described in Fablet *et al.* (2006). We used bowtie (–best) (Langmead *et al.* 2009). We analyzed base composition at each position for piRNAs aligned against *tirant* using SAMStat (Lassmann *et al.* 2011).

To visualize read mapping along *tirant* sequence, we sampled 40,000,000 reads from each small RNA-seq sample using the fastq-sample program from fastq-tools 0.8 (https://homes.cs.washington.edu/∼dcjones/fastq-tools/), and then we selected 23-30 nt-long reads as mentioned above. We used bowtie (–best) (Langmead *et al.* 2009) to map these small RNA-seq reads against our C-subfamily reference *tirant* sequence (see above). We removed the 3′ LTR, so that reads corresponding to LTRs should map at a unique location. We filtered out alignments with a mapq score below 20 using samtools (Li *et al.* 2009). Visualization of the alignments was performed using BamView (Carver *et al.* 2010).

### Cell culture and reporter gene assays

We used reporter gene assays to test the sense and antisense promoter potentials of the LTRs of both subfamilies. LTR sequences were PCR amplified from the Mayotte strain (see Supplementary Material S1 for primer sequences). Primers were designed to include *Kpn*I and *Hin*dIII restriction sites, to allow subsequent directional cloning into the pGL4.10 plasmid (Promega, luciferase reporter gene) using T4 DNA ligase (NEB). S2 *Drosophila* cells were transfected using Cellfectin (Invitrogen) and lyzed for analysis after 24 h of incubation (6 replicates per construct). Luminescence was then estimated using the Dual-Luciferase Reporter Assay System (Promega).

### Genome sequencing and assemblies

Genomic DNA samples were treated with RNAse A at 37° for 30 min and purified using Phenol:Chloroform extraction. The purified genomic DNA samples were fragmented using Covaris S220 sonicator to get fragments of size 300-800 bp. Sequencing libraries were prepared from these fragmented DNA samples using NEBNext UltraTM DNA Library Prep Kit (Illumina, # E7370L). All three libraries were sequenced in one lane of an Illumina HiSeqV4 with 125 bp paired-end reads at the Next Generation Sequencing unit of the Vienna Biocenter Core Facilities (VBCF http://vbcf.ac.at).

Approximately 100 million read pairs (24 Gb) from three accessions (Mayotte, Makindu and Chicharo) were obtained and raw reads were filtered using the unsupervised quality trimming program UrQt (Modolo and Lerat 2015). Contigs were then generated by the Ray assembler v2.3.1 (Boisvert *et al.* 2010) using kmer length varying from 25 to 45. Best assemblies were selected by maximizing both NG50 and the number of contigs larger than 10 kb. For each accession, the assembled genome size was over 100% of the expected genome size (146.7 Mb). The final contig NG50 were 5.38 kb (k = 43), 11.89 kb (k = 39) and 7.36 kb (k = 35), with maximum scaffold length of ∼130 kb, 495 kb and 495 kb for Mayotte, Makindu and Chicharo, respectively. In order to assess completeness of genome assemblies, we searched for conserved genes across arthropods using BUSCO (Simão *et al.* 2015). Among the set of 2,675 genes, 2,254 (84%), 2,403 (90%) and 2,269 (85%) were recovered as complete and single copy in the assemblies of Mayotte, Makindu and Chicharo, respectively.

### Genome and transcriptome analyses

We ran RepeatMasker (Smit *et al.* 2013) on the above assemblies (default parameters, -species Drosophila) and retrieved “Tirant” accessions in the output file in order to identify *tirant* insertion sites. Then, we extracted the flanking sequences for the insertions found above and located them on droSim1 assembly using the BLAT tool (Kent 2002) of UCSC Genome Browser (http://genome.ucsc.edu). We checked that shared *tirant* insertions had the same breakpoints in all strains.

RNA-seq data from ovaries and the corresponding computed read counts for all TE families, including *tirant*, were obtained from Lerat *et al.* (2017) for five strains (Chicharo, Makindu, Mayotte, Zimbabwe, and w501). Differential expression analysis was performed using DESeq2 (Love *et al.* 2014), which model internally corrects for library size, and provides normalization. Read counts obtained for genes on one hand and TEs on the other hand (Lerat *et al.* 2017) were concatenated to make the complete read count table that was analyzed at once using DESeq2.

The *tkv* gene had no assigned *D. simulans* ortholog in the FlyBase gene list that we used for RNA-seq analysis at that time (Lerat *et al.* 2017). In 2018, FBgn0194057 has been assigned as *D. simulans tkv* ortholog (OrthoDB v9.1). Thus, we retrieved the FBgn0194057 sequence, performed the same procedure as for the other genes (Lerat *et al.* 2017), and added the corresponding read counts to our count table.

To visualize read mapping along *tirant* sequence, we sampled 90,000,000 reads from each DNA-seq sample and 25,000,000 reads from each RNA-seq sample (one biological replicate from each strain) to ensure even abundance of reads. In addition, for RNA-seq data, we only used reads longer than 20 nt (PRINSEQ filter, as described above). We used the fastq-sample program from fastq-tools 0.8 (https://homes.cs.washington.edu/∼dcjones/fastq-tools/) and the fastq_sampler.py script (Modolo 2018). Then, we used bowtie2 (–very-sensitive) (Langmead and Salzberg 2012) and mapped DNA-seq and RNA-seq reads against our C-subfamily reference *tirant* sequence (see above). We removed the 3′ LTR, so that reads corresponding to LTRs should map at a unique location. We filtered out alignments with a mapq score below 20 using samtools (Li *et al.* 2009). Visualization of the alignments was performed using BamView (Carver *et al.* 2010).

Transcript structure analysis: we used apytram (apytram v1.1 (Rey *et al.* 2017)) to assemble independently *Hs6st* and *tkv* in Chicharo, Makindu and Mayotte RNA-seq samples (replicates #1). Apytram is an implementation of the Target Restricted Assembly Method (Johnson *et al.* 2013). This method allows to focus on the assembly of only one gene of a transcriptome and not the whole transcriptome. It is based on an iterative process: at the first iteration, a reference gene from another species is used as bait sequences to fish reads in RNA-seq data using BLAST (Camacho *et al.* 2009). Paired reads are used to enlarge this batch of reads. Reads are then *de novo* assembled using Trinity (Grabherr *et al.* 2011), and a new iteration begins with the reconstructed sequences as baits. Here, we used the *Hs6st* and *tkv* genes from *D. melanogaster* as references. This iterative process allows to reconstruct transcripts step by step from homologous regions between *D. melanogaster* and *D. simulans*, and thus non homologous regions can be reconstructed in *D. simulans* transcripts. In addition, if ever *tirant* is exonized in one of the strains, apytram will be able to catch it. Then, to analyze *Hs6st* and *tkv* transcript structures in our samples, we aligned output transcripts on *D. simulans* genome (ASM75419v3.41) using exonerate (Slater and Birney 2005). Finally, we manually checked the hit distribution against *D. simulans* genome to search for the presence of *tirant* in transcript sequences. We used TopHat2 (Kim *et al.* 2013) and ggsashimi (Garrido-Martín *et al.* 2018) to view transcript structures using sashimi plots.

### ChIP assays

Biological duplicates were produced. For each replicate, seventy ovary pairs were dissected from three to five day-old females and flash frozen to maintain chromatin integrity. Chromatin extraction and sonication were carried out as described in Akkouche *et al.* (2013) with the following modifications: 30 sonication cycles (30 sec on and 30 sec off on high) were performed to obtain chromatin fragments comprised between 200 and 500 bp. The chromatin fragments were then aliquoted and diluted into six IP samples of 500 µL along with a 10% Input sample. IP samples were then immunoprecipitated following the manufactor’s protocol (Magna ChIP, Millipore) with 3 µg of antibodies (H3K4me3: Abcam # ab8580; H3K9me3: Actif Motif # 39161).

Quantitative PCR (qPCR) was used to estimate IP enrichment as previously described (Fablet *et al.* 2009; Akkouche *et al.* 2013) and performed as technical triplicates. For each of the studied *tirant* insertions, we used qPCR primers located at the insertion site and ∼2 kb away, in both directions. At the insertion site, we designed primer pairs displaying one primer within *tirant* and the other primer in the flanking sequence. For strains devoid of *tirant* insertion, we used the same primer in the flanking region as mentioned above and designed a second primer in the flanking region beyond the site of *tirant* insertion. These two primer pairs allow us to amplify *tirant* insertion sites in strains where *tirant* is present as well as in strains were *tirant* is absent. Primer sequences are provided in Supplementary Material S1. As controls, we used *light* for H3K9me3 enrichment (Fw: 5′-GCT AGG CAA TGA CAA AGT CCT TTG GG-3′ and Rv: 5′-GCA TTC GTC TGA AGT CGG CAG ATA G-3′), and *rpl32* for H3K4me3 enrichment (Fw: 5′-CAG CTT CAA GAT GAC CAT C-3′ and Rv: 5′-GTT CGA TCC GTA ACC GAT GT-3′). Raw data from qPCRs are provided in Supplementary Material S2.

### Long PCR

We PCR amplified *tirant* insertion at the *Hs6st* locus using DreamTaq (Fermentas), with a 7 min elongation time and a 68° hybridization. Primer sequences are 5′- GCAACACTGACAGCAACTACA -3′ and 5′- TCCTTGCTAGCTACATGGAAC -3′.

### Data Availability

Genomic raw reads are available under the SRA accession number SRP128969. Genome assemblies, modified versions of TEcount.py and the list of TE sequences are available at ftp://pbil.univ-lyon1.fr/pub/datasets/Fablet2018. SupplMat S1 contains primer sequences. SupplMat S2 contains ChIP qPCR results. SupplMat S3 is an alignment of reference C and S sequences. Alignments of piRNAs against *tirant* reference sequence are provided Figure S1. Figure S2 displays graphics of piRNA base composition. Gels obtained from PCR products are shown Figure S3. Sashimi plots for *tkv* and *Hs6st* splicing are provided Figure S4. Supplemental material available at Figshare: https://doi.org/10.25387/g3.7578380.

## Results

### Tirant is differentially expressed across wild-type strains

**Figure 1:**
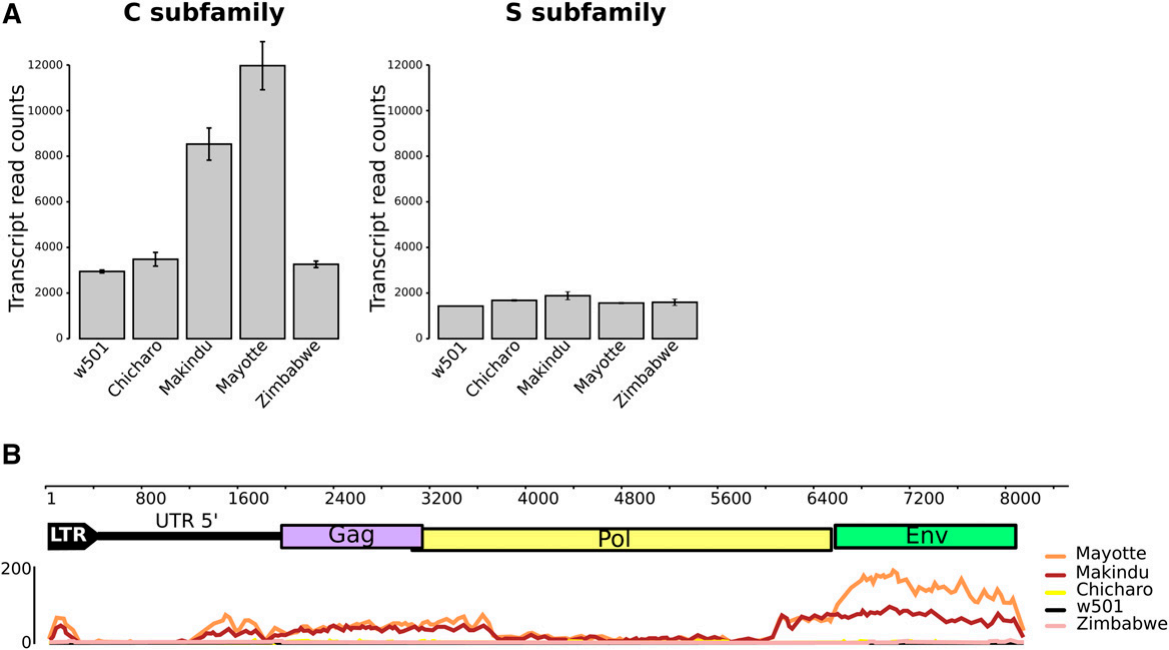
*Tirant* trancripts analyzed from RNA-seq data. A. Tirant transcript normalized read counts. B. Mapping of tirant reads against the reference tirant copy (subfamily C). The upper part is tirant structure, to scale (in bp). The lower part is read coverage along tirant sequence, obtained from samples of 25,000,000 paired-end reads. We removed LTR 3′ to get rid of multi-mapping issues.

Using RNA-seq data obtained from ovary samples, we were able to quantify *tirant* transcript levels in *D. simulans* strains (Figure 1). As expected (Fablet *et al.* 2006, 2009), subfamily S transcript levels were found at background level in all strains, and so were subfamily C transcript levels in w501, Chicharo and Zimbabwe strains. *Tirant* produces significant amounts of transcripts only in Makindu and Mayotte strains (Figure 1A). The mapping of *tirant* reads against the reference sequence shows enrichment in the *env* region (Figure 1B). This pattern could reflect either the existence of a master copy deleted for *gag* and *pol*, or the prevalence of splicing events of the *gag* and *pol* ORFs, as described by Marsano *et al.* (2000) in *D. melanogaster*. The higher abundance of *env* reads in the Mayotte strain is in agreement with the production of the *tirant* Env protein in this strain, which we previously observed using immunostaining (Akkouche *et al.* 2012).

### Tirant displays specific sense and antisense piRNAs

**Figure 2:**
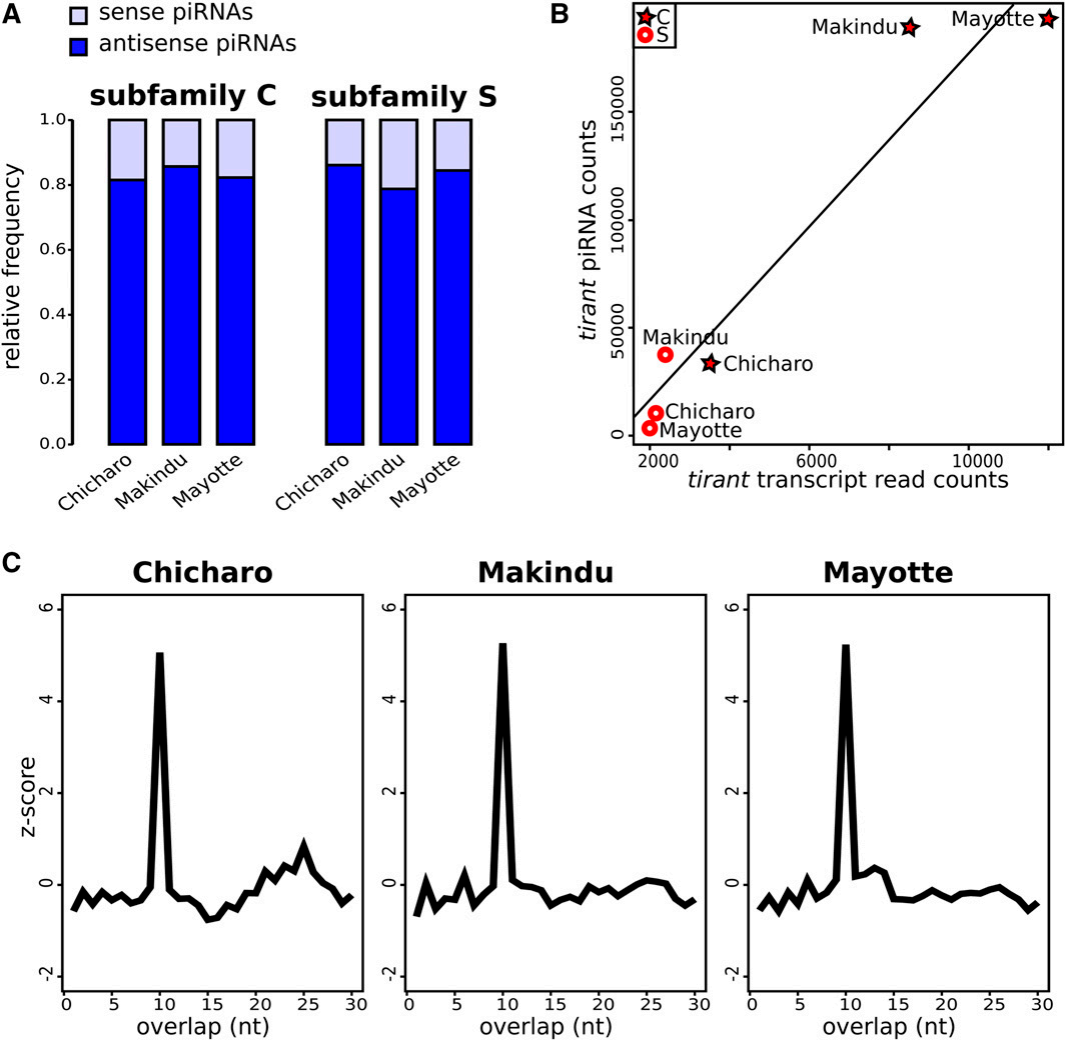
*Tirant*-specific piRNAs. A. Relative proportions of sense and antisense piRNAs. B. Positive correlation between tirant transcript normalized read counts and tirant piRNA read counts in wild-type strains. C. Ping-pong signatures: there is a clear enrichment in 10 nt overlaps.

We specifically analyzed small RNAs for the two strains that show significant *tirant* transcript levels (*i.e.*, Makindu and Mayotte), and one strain presenting background levels (*i.e.*, Chicharo). We found sense and antisense *tirant*-specific piRNAs, corresponding to both the C and S subfamilies. Sequence similarity between subfamilies C and S varies along *tirant* sequence: it is the lowest in the UTR 5′ region (∼65% (Fablet *et al.* 2006)) and displays higher values within coding sequences (76–85% global similarity rate (Fablet *et al.* 2009)). Although we cannot exclude that some reads may be mis-attributed to the other subfamily, it should not happen for the majority. Except for a peak in the LTR region, *tirant* piRNAs evenly map along *tirant* reference sequence in all three strains (Figure S1). *Tirant* piRNAs displayed comparable proportions of antisense piRNAs to total piRNAs in all three strains and *tirant* subfamilies, ranging from 0.798 to 0.869 (Figure 2A). In addition, we looked for ping-pong signatures, which are 10 nt overlaps between sense and antisense piRNAs (Brennecke *et al.* 2007). We found clear, similar enrichments for 10 nt overlaps in all three strains (Figure 2C), indicative of similar, functional, secondary piRNA pathways against *tirant* in all three strains. In addition, these piRNAs showed expected enrichment in 1U and 10A (Figure S2) (Saito *et al.* 2006; Brennecke *et al.* 2007).

Moreover, our data allowed us to reveal a positive correlation between *tirant* piRNA read counts and *tirant* transcript read counts (Pearson correlation, r = 0.963, p-value = 0.002, Figure 2B). The correlation remains strong using only sense (Pearson correlation, r = 0.965, p-value = 0.002), or antisense (Pearson correlation, r = 0.946, p-value = 0.004) piRNAs.

### Tirant LTRs display antisense promoter activity

**Figure 3:**
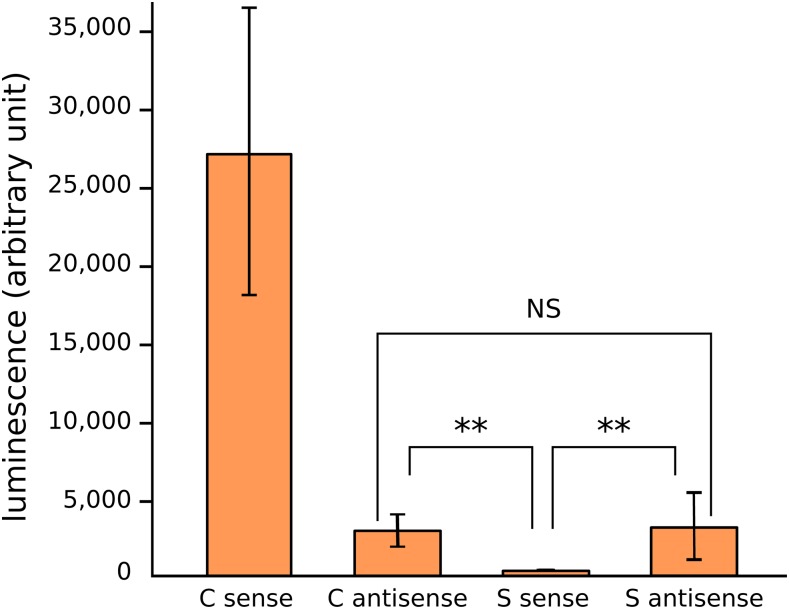
*Tirant* LTR promoter activity. LTRs of C and S subfamilies in sense and antisense orientations were cloned upstream of a luciferase reporter gene and tested for promoter activity in S2 cells.

As new TE insertions may behave as dual-strand piRNA clusters (Mohn *et al.* 2014; Shpiz *et al.* 2014), we wondered whether *tirant* copies could be able to produce antisense piRNA precursors. To investigate such hypothesis, we tested the C and S LTRs for their sense and antisense promoter abilities. We known from a previous work that sequence similarity between C and S LTRs is 65% on average, while sequence similarity within each subfamily is higher than 98% (Fablet *et al.* 2006). It appears that the different constructs behave significantly differently regarding gene expression promotion (Kruskal Wallis test, df = 3, p-value = 2.10^−4^), with LTR C sense being the strongest promoter and LTR S sense being the weakest. In addition, we find that both subfamily LTRs display weak antisense promoter activity (Figure 3) (Wilcoxon tests, C antisense *vs.* S sense: p-value = 0.002, S antisense *vs.* S sense: p-value = 0.002, C antisense *vs.* S antisense: p-value = 0.485). Therefore, the results suggest that, although subfamily S is not significantly involved in the production of *tirant* transcripts, and albeit the weak activity observed *in vitro*, it may rather be involved in the production of antisense piRNA precursors.

### Tirant displays insertional polymorphism

The influence of a TE on the host genome depends on its insertion sites. To investigate *tirant’s* impacts, we produced genome assemblies for Chicharo, Makindu and Mayotte, and used RepeatMasker (Smit *et al.* 2013) to retrieve *tirant* insertion sites. We excluded scaffolds exclusively made of *tirant*. Nested *tirant* insertions into other TE sequences were also excluded since we are not able to locate them precisely on the genome. The final number of retrieved *tirant* insertions is congruent with our knowledge of *tirant* copy numbers (Fablet *et al.* 2006, 2009; Akkouche *et al.* 2012), indicating that we did not missed a significant number of loci.

Overall, we localized precisely 15 insertions, the majority of which are unique to one strain (Table 1). Among these, six are located in introns and two upstream transcription start sites (TSSs), which are the insertions the most likely to have an impact on genes. Insertion #10 is located 242 bp upstream of *CanA1* TSS, and insertion #11 533 bp upstream of *Lamp1* TSS. We identified five additional insertions, attributed to the U chromosome (Unknown chromosome), and which likely correspond to heterochromatic regions. Among these, one is shared across strains.

**Table 1:** *Tirant* insertion sites as identified using RepeatMasker on genome assemblies. +: insertion found in the genome assembly of the strain. Due to the fragmented nature of our assemblies, we are not able to interpret “absence of detection” as “absence of tirant”. These data do not distinguish tirant subfamilies. TSS: Transcription Start Site. U: Unkown chromosome. ≥ LTR: we detected one LTR (417 bp) at the extremity of a scaffold, which means that we do not know whether the insertion is a solo LTR or longer. Insertion #10 is located 242 bp upstream of CanA1 TSS, and insertion #11 533 bp upstream of Lamp1 TSS. D. simulans orthologs as assigned in OrthoDB v9.1 (retrieved via FlyBase in november 2018) are mentioned in brackets and italics.

Insertion #	Chr	Annotation	Insertion size (bp)	Mayotte	Makindu	Chicharo
1	3R	Intron CG34383 (*FBgn0190438*)	41	+	+	+
2	3L		102	+		
3	3R	Intron *Hs6st* (*FBgn0041661*)	≥ LTR	+		
4	2L	Intron *vri* (*FBgn0194720*)	≥ LTR	+		
5	3R	Intron CG11873 (*NA*)	≥ LTR	+		
6	3R		> 95	+		
7	2L		446	+	+	
8	3h		490	+		
9	2h		> 589	+		
10	3R	Near *CanA1* TSS (*FBgn0192983*)	> 96	+		
11	2L	Near *Lamp1* TSS (*FBgn0195671*)	704	+	+	+
12	2L	Intron *tkv* (*FBgn0194057*)	≥ LTR		+	
13	2L		> 100		+	
14	2R		> 95		+	
15	2R	Intron CG17684 (*FBgn0268563*)	8014			+
16	U		> 99	+	+	+
17	U		>98		+	+
18	U		115	+		
19	U		> 93	+		
20	U		438			+

It is to be noted that, following this procedure, the *tirant* insertion into the *tkv* gene (insertion #12) is only found in the Makindu genome, while we have experimental evidence that it also exists in the Mayotte strain. This stresses on the non exhaustive nature of this approach, which strongly relies on scaffold assemblies. Indeed, we used a PCR approach to confirm *tirant* insertion polymorphism for insertions #3 and #12, which are studied in more details below. (See Supplementary Material S1 for primer sequences). For insertion #3, PCR profiles were congruent with *tirant* being present only in Mayotte and absent in Makindu and Chicharo (Figure S3A). For insertion #12, PCR profiles indicated *tirant* presence in Makindu and Mayotte, and *tirant* absence in Chicharo (Figure S3B). However, we could also detect a very faint band indicating *tirant* absence in Makindu. This suggests that this insertion may be heterozygous or even absent in few individuals.

**Figure 4:**
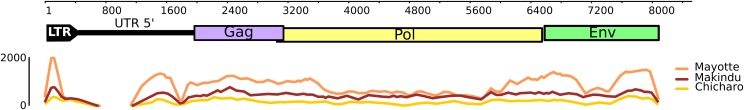
Mapping of genomic reads against *tirant* reference sequence. The upper part represents tirant structure, to scale (in bp). The lower part depicts read coverage along tirant sequence (subfamily C), obtained from samples of 90,000,000 paired-end reads. We removed LTR 3′ to get rid of multi-mapping issues.

Accessing the precise structure of each *tirant* insertion is difficult without long reads. Therefore, we mapped genomic reads against our *tirant* reference sequence to have an idea of the representativeness of each region within the genomes of our strains. We found that reads map evenly along the reference sequence for Chicharo and Makindu, suggesting the presence of full-length copies (Figure 4). Mayotte displays the highest number of reads after normalization, in agreement with a higher number of *tirant* copies. We note a slight depression in the center of the *pol* and *env* ORFs (Figure 4), suggesting the existence of internally deleted copies. The drop of mapping coverage within the 5′ UTR region is due to our mapq filter (see Material and Methods), which prevents multi-mapping against repeated regions, such as the minisatellite that is described within *tirant* 5′ UTR (Fablet *et al.* 2006).

### Mild impact of tirant on neighboring genes

**Figure 5:**
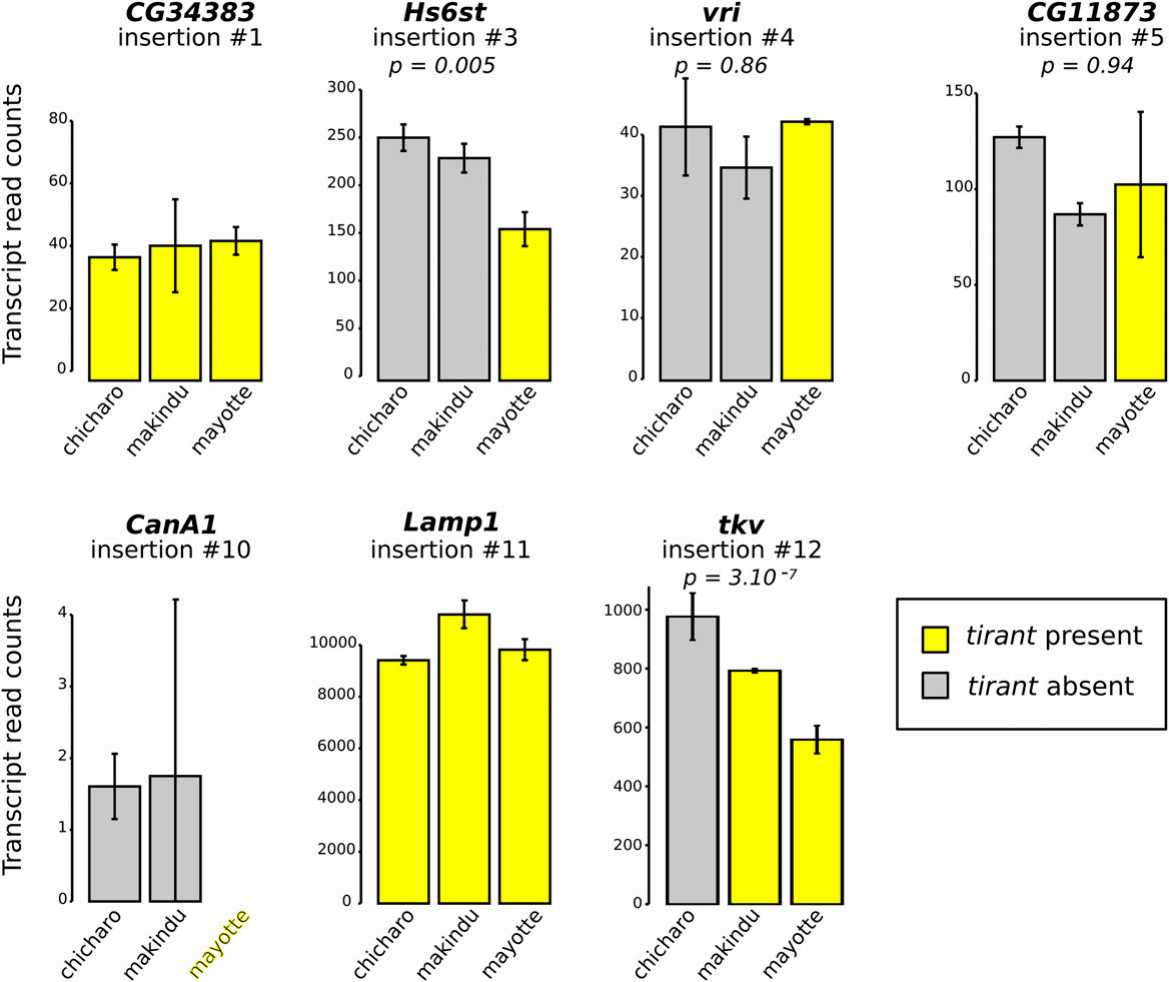
RNA-seq results for genes at or near *tirant* insertions. Normalized read counts. Please refer to Table 1 for tirant insertion details. Error bars are standard deviations on biological duplicates. When tirant insertion is polymorphic, we provide adjusted p-values comparing strains with and without tirant insertion, as calculated by DESeq2. Gene CG17684 (insertion #15) has no D. simulans ortholog in the list of genes used for RNA-seq data mapping.

For those insertions located near or within genes (within introns), we tested whether *tirant* could have an effect on gene expression through modifications of chromatin structure. To do so, we used data produced from ovaries, and we focused on the insertions of *tirant* that fulfill the following criteria: i) the gene into which *tirant* is inserted displays a high enough number of reads in our RNA-seq dataset (> 100 reads), ii) its expression pattern is contrasted across strains with and without the *tirant* insertion (Lerat *et al.* 2017). We found only two of such insertions (Figure 5). The two insertions of interest are located within the *Hs6st* and *tkv* genes, respectively (insertions #3 –only in Mayotte– and #12 –in Mayotte and Makindu– in Table 1, respectively). In both cases, gene transcript levels are lower for strains where *tirant* is present compared to strains devoid of *tirant* at the considered locus (DESeq2 padj values: *Hs6st*: Chicharo *vs.* Mayotte: 0.0157, Makindu *vs.* Mayotte: 0.0916; *tkv*: Chicharo *vs.* Makindu: 0.0617, Chicharo *vs.* Mayotte: 2.10^−8^). Moreover, FlyBase *D. simulans* genome annotation indicates the presence of additional genes within *Hs6st* (FBgn0190828, FBgn0270486, FBgn0190826, and FBgn0269631) and *tkv* (FBgn0270637, FBgn0194717, FBgn0270509, and FBgn0194718) introns. However, virtually none of the reads of our data sets map against these sequences.

**Figure 6:**
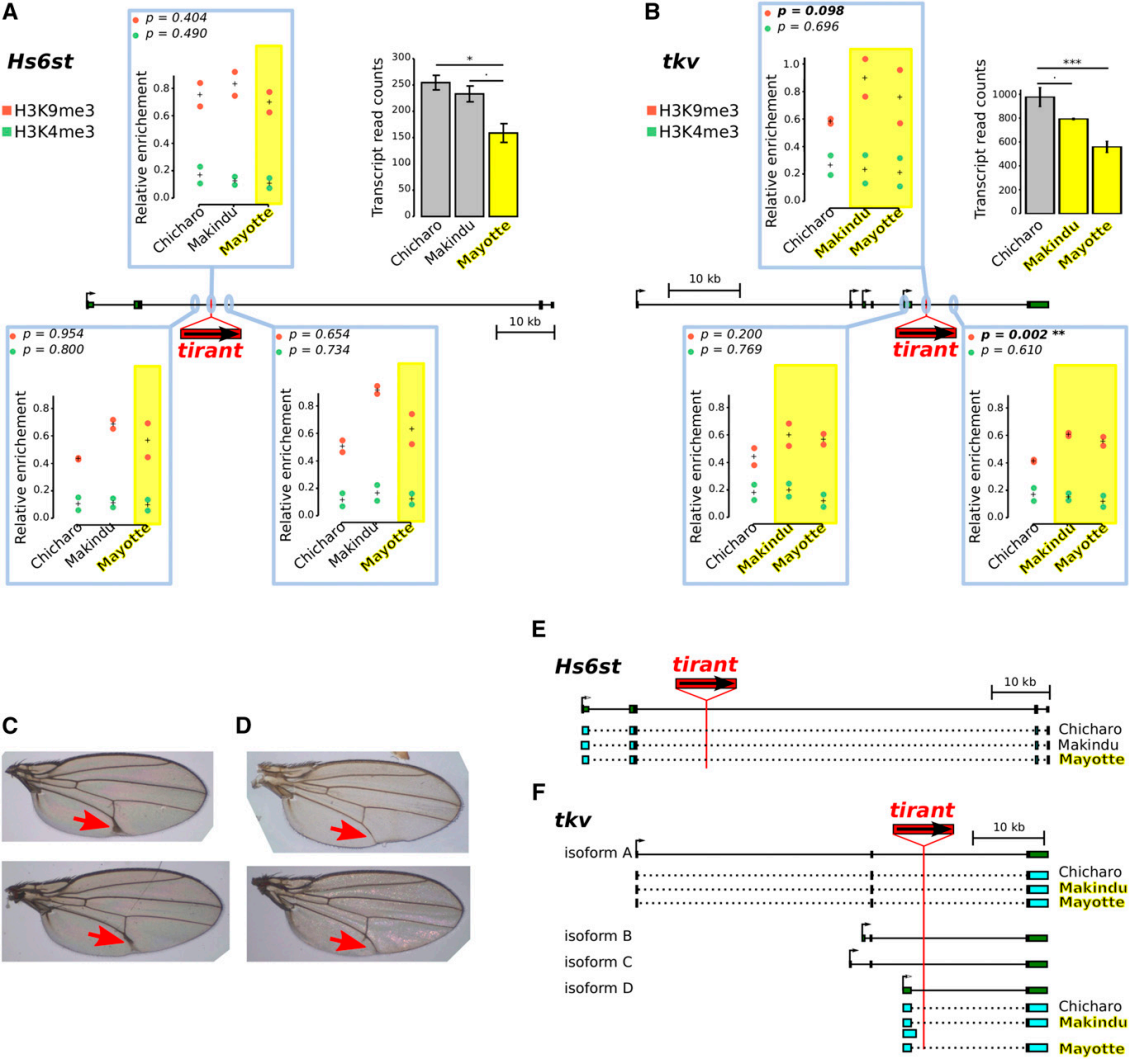
Analysis of two *tirant* insertions within introns. A. and B. Histone mark enrichments at two tirant insertion sites. P-values in the upper left corners are produced from t-tests between strains with the tirant insertion (yellow) and strains without tirant (uncolored). Left panel: A. tirant insertion #3 into Hs6st, right panel B.: tirant insertion #12 into tkv. Genes are drawn to scale in the middle of each panel; thin lines: introns, thick boxes: exons. H3K4me3 and H3K9me3 enrichments are shown in green and red, respectively, and quantified relative to control genes (see Material and Methods section). They are displayed in boxes corresponding to the location of the qPCR amplicons: either at the tirant insertion site or 2 kb away. Biological duplicates were produced for ChIP experiments. “+” indicates the mean between biological replicates. Normalized transcript levels for the considered genes are provided in the upper right corners (as already shown Figure 5). Error bars are standard deviations. Strains in which tirant is present at the considered locus are highlighted in yellow. Statistical significance for RNA-seq results was assessed by padj values provided by the DESeq2 analysis. Padj values: Hs6st: Chicharo *vs.* Mayotte: 0.0157, Makindu *vs.* Mayotte: 0.0916, tkv: Chicharo *vs.* Makindu: 0.0617, Chicharo *vs.* Mayotte: 2.10^−8^. C. and D. Vein phenotype in the Mayotte strain. C. We observed thick L5 veins (red arrows) in seven out of 97 flies in the Mayotte strain. D. Wild-type vein phenotype observed in 90 out of 97 flies in Mayotte (the same phenotype is also observed in all examined flies in Makindu and Chicharo). E. and F. Transcript structures. Reference structures from D. melanogaster, as retrieved from the UCSC Genome Browser, are depicted in dark green, thin lines: introns, thick boxes: exons. Reconstructed exons are shown in light blue. E. Hs6st transcript structures. All three strains display the same transcript structure as described in D. melanogaster. F. tkv transcript structures. We observe a Makindu-specific transcript of isoform D.

We investigated chromatin structure to look for epigenetic modifications that could spread on neighboring regions. We performed ChIP experiments on H3K4me3 and H3K9me3. The former is characteristic of open chromatin conformation, particularly found at promoters; the later is the heterochromatic mark known to be involved in TE transcriptional silencing. We used qPCR primers located at the insertion site and ∼2 kb away, in both directions (Figure 6A, 6B). Primers at the insertion sites were the same as those mentioned above to detect *tirant* presence (Supplementary Material S1). No obvious effect of *tirant* presence on either chromatin mark at the *Hs6st* gene is observed: Mayotte enrichment levels (*tirant* present) lie in between Chicharo and Makindu levels (*tirant* absent). On the contrary to *Hs6st* insertion, it appears that *tirant* neighborhood is moderately enriched in H3K9me3 at the *tkv* insertion site compared to strains without *tirant* (fold change = 1.43 ; *t*-test, p-value = 0.098). In addition, these histone post-translation modifications spread at least 2 kb away downstream *tirant* (fold change = 1.41 ;*t*-test, p-value = 0.002). Regarding H3K4me3 enrichments, we could not detect differences between strains with and without *tirant* at the *tkv* locus.

Since *tkv* mutations are known to alter wing vein phenotypes (Lindsley and Zimm 1992), we examined wing morphologies in our flies (we did not separate sexes). We found seven flies out of 97 in the Mayotte strain which displayed thick L5 veins (Figure 6C, 6D), while we did not find any out of 100 Makindu flies nor 100 Chicharo flies (Fisher exact test, p-value = 0.014). It is to be noted that Mayotte is the strain that shows the strongest reduction in *tkv* transcript levels.

As it was recently shown that piRNAs may also regulate TEs through splicing (Teixeira *et al.* 2017), we analyzed transcript structures in *Hs6st* and *tkv*. *Hs6st* displays virtually no transcript isoform variation in the reference *D. melanogaster* genome. Unsurprisingly, we found the described *Hs6st* transcript to be produced in all three strains (Figure 6E). In contrast, four *tkv* isoforms are described in *D. melanogaster*. Isoforms B and C are not found to be produced in our samples, and isoform A displays the same reference structure in all three strains. Variability was observed regarding isoform D: the first exon is 1,371 bp longer in the Makindu strain compared to the others (Figure 6F). Sashimi plots are provided Figure S4. Interestingly, this variable splice site is located less than 800 bp from *tirant* insertion in Makindu and Mayotte, and thus in the region where we find chromatin structure to be modified in association with *tirant* insertion. It is thus tempting to speculate that *tirant*-induced chromatin modifications may affect splice site definition. In addition, we note that we do not find evidence of *tirant* exonization neither in *Hs6st* nor in *tkv*.

## Discussion

### The repertoire of tirant-specific piRNAs

Our in-depth analysis of *tirant*-specific small RNAs revealed the presence of both sense and antisense piRNAs. While the primary piRNA pathway exclusively produces antisense piRNAs, the secondary piRNA pathway leads to the production of both sense and antisense piRNAs, characterized by the so-called ping-pong signature (Brennecke *et al.* 2007). Thus, the detection of sense *tirant* piRNAs indicates that the secondary pathway is involved in *tirant* control, as already proposed by previous experimental analyses (Akkouche *et al.* 2013). This is reinforced by the identification of ping-pong signatures, which are similar across strains. However, we cannot exclude the involvement of the primary pathway as well, especially considering the high proportions of antisense *tirant*-specific piRNAs.

So far, we do not know which piRNA clusters are involved in the production of *tirant*-specific piRNAs in *D. simulans* strains; however, our observation of antisense promoter properties for both subfamilies, albeit weak, indicates that some copies may have the ability to behave as dual-strand piRNA clusters, as proposed by two research studies (Mohn *et al.* 2014; Shpiz *et al.* 2014). While the S subfamily is not transcribed and apparently not involved in *tirant* activity, this potential role in piRNA precursor production may also explain why it is found to be conserved across strains (Fablet *et al.* 2006). However, although we provide *in vitro* evidence that some *tirant* LTR sequences may behave as antisense promoters, we do not know whether the antisense *tirant* piRNAs that we detect are indeed produced from these promoters or from promoters outside of the element itself.

Moreover, while we observed significant differences in *tirant* piRNA amounts across strains, we could not detect differences in sense *vs.* antisense piRNA ratios neither in ping-pong signatures, indicating that *tirant* control by the piRNA pathway may take place the same way and with comparable efficiencies in the three considered strains.

### Dynamics of tirant control by piRNAs

Our survey in wild-type strains revealed a positive correlation between the amounts of *tirant* transcripts and *tirant* piRNAs in ovaries in normal conditions. This is in agreement with what we found using all TE families in the same strains (Lerat *et al.* 2017). This is also congruent with data obtained by others from *D. melanogaster* laboratory strains in normal conditions (Kelleher and Barbash 2013), and in accordance with the accepted model claiming that TE transcripts fuel the ping-pong loop (Senti *et al.* 2015).

Nevertheless, these results contrast with what was found in other studies. Notably, in a previous work, in order to understand *tirant* control, we performed crosses between Makindu and Chicharo, because they display contrasted *tirant* copy numbers and activities (Akkouche *et al.* 2013). When the mother comes from Makindu and the father comes from Chicharo, *tirant* is properly controlled in the progeny. This direction of cross is called “RT” (Regulated Tirant). However, when the reciprocal cross is performed, *tirant* shows a strong accumulation of transcripts in the somatic follicle cells of the progeny’s ovaries (Akkouche *et al.* 2013). This direction of cross is called “NRT” (Non-Regulated Tirant). This disruption of *tirant* control is associated with a lack of *tirant*-specific piRNAs, while these piRNAs remain relatively abundant in the RT direction of cross (Akkouche *et al.* 2013). It results in a negative correlation between *tirant* transcript amounts and *tirant*-specific piRNA amounts.

We propose that the nature of the correlation between TE transcripts and piRNAs reflects genome stability. In normal conditions – which corresponds to a balanced relationship between the genome and its TEs, the correlation is positive. When the equilibrium is broken – and so far we do not know which sensor could detect such event –, the correlation becomes negative. Such patterns were also observed in the case of the *I* non-LTR retrotransposon in *D. melanogaster* strains (Chambeyron *et al.* 2008). Indeed, Chambeyron *et al.* described that strain JA has no *I* transcripts nor *I*-specific piRNAs while strain HT2 displays high amounts of both *I* transcripts and piRNAs (Chambeyron *et al.* 2008). This corresponds to the positive correlation we propose at equilibrium. On the other hand, when dysgenic crosses are performed and disrupt genome / *I* stable relationship, an opposite correlation is observed: SF females –which suffer from sterility– accumulate *I* transcripts due to a lack of *I* piRNAs; on the contrary, RSF females –in which fertility is restored– lower *I* transcript levels due to a high production of *I* piRNAs. Thus, the results obtained from *tirant* in the present work may reflect a behavior common to all TE families.

### Tirant’s soft influence on neighboring genes

In most organisms, TE control is mainly achieved by epigenetic mechanisms (Slotkin and Martienssen 2007; Siomi *et al.* 2011). In *D. melanogaster*, these essentially consist in piRNAs and H3K9me3 (Saito *et al.* 2006; Aravin *et al.* 2007; Sienski *et al.* 2012; Le Thomas *et al.* 2013). We know that this heterochromatic mark may spread to flanking regions up to a 5 kb distance (Rebollo *et al.* 2011). Rebollo *et al.* used mouse strains and did not find that heterochromatin spreading recurrently affected gene activity, probably due to the fact that such TE insertions would be too deleterious (Rebollo *et al.* 2011). In a *Drosophila* system, Sienski and colleagues also observed such spreading, which in addition did impact neighboring genes in mutants in cell cultures (Sienski *et al.* 2012). In the present study, we used the *tirant* system to investigate the spreading of TE chromatin marks in a natural context. We found a large level of insertion polymorphism across the strains we investigated (Table 1). We identified two cases, corresponding to insertions into introns of the *Hs6st* and *tkv* genes, associated with changes in gene transcript levels. While we could not detect any effect of *tirant* insertion on chromatin structure in the case of *Hs6st*, *tirant* insertion into the *tkv* gene was associated with a moderate enrichment in H3K9me3 marks at the insertion site and up to 2 kb away from the insertion site, compared to the strain devoid of *tirant*. In addition, we noted that the *tirant* effect that we detect in Makindu may be underestimated since there may be heterozygosity or absence of the insertion in some individuals. We searched for other TEs in the genome assemblies of all strains in a ∼10 kb window around this particular *tirant* insertion, and could not find any. This suggests that the effects observed on chromatin structure and gene expression may be attributed to the insertion of *tirant*. Moreover, we observed a thick veins phenotype in 7% of Mayotte flies while we could not observe it in Makindu nor Chicharo flies. Mayotte is the strain displaying the strongest reduction in *tkv* transcript levels (Mayotte *vs.* Chicharo: log2FC = -0.80). Although we cannot exclude other differences in genetic backgrounds, we propose that the *tirant* insertion into *tkv* in Mayotte may be responsible for this thick veins phenotype, through *tkv* expression reduction induced by heterochromatin formation. Nevertheless, variation in *tkv* transcript levels is observed –probably associated with micro-environmental variability–, which may explain why most flies do not show the phenotype.

In addition, as it was recently shown that piRNAs may also regulate TEs through splicing (Teixeira *et al.* 2017), we analyzed transcript structures in *Hs6st* and *tkv*. We found no effect of *tirant* insertion in *Hs6st* transcript structures. This observation parallels the absence of *tirant* effect on *Hs6st* chromatin conformation. In contrast, we observed variation in *tkv* splice sites nearby *tirant* insertion in the Makindu strain: the first exon of isoform D is 1,371 bp longer than expected. It is tempting to speculate that *tirant*-induced chromatin modifications disrupt splice site definition; however, we do not find the same pattern in the Mayotte strain, which also displays *tirant* insertion. Further investigation is needed at a larger scale to determine the impacts of TEs on splice site definition.

*Tirant* insertions have either no or moderate effects on nearby gene expression. The insertion into *tkv* may have an impact on chromatin structure and gene expression whereas the *tirant* insertion into *Hs6st* does not. Our results are congruent with the recently published study of Lee and Karpen, although regarding a different histone mark. Based on a *D. melanogaster* genome-wide study, they report the spreading of TE-associated H3K9me2 marks on regions flanking the insertions and an effect on the expression level of flanking genes only for about half of the TE insertions (Lee and Karpen 2017). So far, we have not identified which properties of a TE insertion make it affect nearby genes or not. We know from a previous work that *tirant* is full length at the *tkv* locus in the Makindu strain (Fablet *et al.* 2009). We performed a long PCR at the *Hs6st* locus and observed a band congruent with a full-length insertion as well (Figure S3C). In addition, both *tirant* sequences are oriented the same way as the gene. We may note that the *Hs6st* locus displays insertion polymorphism for helitron *N1_Dbi*, which is found approximately 1 kb away from *tirant* in Chicharo and Mayotte but not in Makindu. Apart from this helitron and *tirant*, no other TE sequences are found within a 4 kb window around *tirant* insertion site. To sum up, we still do not know why *tirant* affects *tkv* but not *Hs6st*.

*Tirant* effects are of small size: on average, a 30% reduction in *tkv* transcript amounts and an increase of 33–42% in H3K9me3 enrichment. If it were not a small effect, we speculate that the insertion would be too deleterious and removed from the genome by natural selection. In addition, cases were also reported in which TE insertions had only a modest impact on neighboring genes in standard conditions, but significant effects under stress conditions (Naito *et al.* 2009).

### Conclusion

In the present study, we use natural variability regarding the *tirant* ERV of *D. simulans* to identify potential impacts of TEs on the host genome. We show that, to a moderate extent, a *tirant* insertion may spread heterochromatin to flanking regions where genes lie, and be associated with transcript level reduction for the gene, with potential phenotypic impacts. Our results also illustrate that TE effects on the genome may not be as pervasive as recently proposed (Lee and Karpen 2017) since we detected them only for one insertion and not for the others. In addition, our data suggest the existence of a dynamic relationship between TE transcripts and the piRNAs that control them. This is illustrated by a positive correlation between these two variables in normal conditions, which is reverted to a negative correlation in the case of dysgenic crosses. We speculate this may happen when asymmetry is too high between ping-pong partners abundances, –*ie* maternally transmitted piRNAs on one hand and TE transcripts on the other hand, for the piRNA pathway to be efficient.

Taken together, our results participate in the understanding of the equilibrium between the host genome and its TEs. This study opens the way to the investigation of the transition from the equilibrium disruption to a restored balance between the genome and its TEs, which are fundamental aspects of the understanding of TE biology and genome stability.
